# Altered local gyrification and functional connectivity in type 2 diabetes mellitus patients with mild cognitive impairment: A pilot cross-sectional small-scale single center study

**DOI:** 10.3389/fnagi.2022.934071

**Published:** 2022-09-20

**Authors:** Pengfei Shao, Xin Li, Ruomeng Qin, Hengheng Xu, Xiaoning Sheng, Lili Huang, Junyi Ma, Yue Cheng, Haifeng Chen, Bing Zhang, Hui Zhao, Yun Xu

**Affiliations:** ^1^Department of Neurology, Affiliated Drum Tower Hospital, Nanjing University Medical School, Nanjing, China; ^2^Jiangsu Key Laboratory for Molecular Medicine, Nanjing University Medical School, Nanjing, China; ^3^Jiangsu Province Stroke Center for Diagnosis and Therapy, Nanjing, China; ^4^Department of Radiology, Nanjing Drum Tower Hospital Clinical College of Nanjing Medical University, Nanjing, China; ^5^Department of Radiology, Affiliated Drum Tower Hospital, Nanjing University Medical School, Nanjing, China; ^6^Department of Neurology, Affiliated Taikang Xianlin Drum Tower Hospital, Nanjing University Medical School, Nanjing, China

**Keywords:** type 2 diabetes mellitus, gyrification, resting-state functional connectivity, mild cognitive impairment, MRI

## Abstract

**Aims:**

This research aimed to explore alterations in the local gyrification index (GI) and resting-state functional connectivity (RSFC) in type 2 diabetes mellitus (T2DM) patients with mild cognitive impairment (MCI).

**Methods:**

In this study, 126 T2DM patients with MCI (T2DM-MCI), 154 T2DM patients with normal cognition (T2DM-NC), and 167 healthy controls (HC) were recruited. All subjects underwent a battery of neuropsychological tests. A multimodal approach combining surface-based morphometry (SBM) and seed-based RSFC was used to determine the structural and functional alterations in patients with T2DM-MCI. The relationships among the GI, RSFC, cognitive ability, and clinical variables were characterized.

**Results:**

Compared with the T2DM-NC group and HC group, T2DM-MCI patients showed significantly reduced GI in the bilateral insular cortex. Decreased RSFC was found between the left insula and right precuneus, and the right superior frontal gyrus (SFG). The altered GI was correlated with T2DM duration, global cognition, and episodic memory. The mediation effects of RSFC on the association between GI and cognition were not statistically significant.

**Conclusion:**

Our results suggest that GI may serve as a novel neuroimaging biomarker to predict T2DM-related MCI and help us to improve the understanding of the neuropathological effects of T2DM-related MCI.

## Introduction

The global prevalence of type 2 diabetes mellitus (T2DM) has increased in the past 50 years (Bellary et al., 2021). Previous studies have indicated that T2DM is associated with an increased risk of mild cognitive impairment (MCI) involving various cognitive domains, such as attention, executive function, and memory (Li, 2015; Li et al., 2019). MCI is defined as a slight cognitive dysfunction that is measurable in cognitive tests but does not affect daily living yet (Petersen et al., 2018). However, T2DM promotes the progression of MCI to more severe stages, such as dementia (Cukierman et al., 2005). Therefore, early identification and detection of alterations in T2DM patients with MCI may help clinicians take early prevention measures.

Magnetic resonance imaging (MRI) has been shown to be a valuable tool for investigating the structural and functional changes in the brains of patients with T2DM. To date, the majority of structural MRI studies have focused on the volumes in specific brain regions using the voxel-based morphometry (VBM) approach (Rosenberg et al., 2018). However, compared to the traditional VBM method, surface-based morphometry (SBM) could improve the reliability of cortical analyses owing to its increased spatial acuity in the processing step (Coalson et al., 2018). Furthermore, VBM results could be partly driven by differences in surface-based measures (e.g., cortical thickness and gyrification) (Hutton et al., 2009). Therefore, an increasing number of studies are using the SBM approach to study various neurological disorders (e.g., Alzheimer's disease and Parkinson's disease) (Sterling et al., 2016; Nunez et al., 2020).

Gyrification index (GI), the degree and pattern of cortical folding, is an important and distinguishing characteristic of the human cerebral cortex. The greater GI means the larger neuron number and the lower communication costs of the cortex within limited space (Gautam et al., 2015). Moreover, GI has been suggested to be specifically influenced by genetic and environmental factors (Zilles et al., 2013). A study reported that regular meditation practice might alter the GI of specific brain regions, suggesting that lifestyle factors may have a modulating impact on gyrification. Another study enrolling 70 patients with Parkinson's disease (PD), suggested the accelerated decrease of GI occurs shortly after PD diagnosis and becomes prominent in the later stages (Sterling et al., 2016). Therefore, GI could reflect ongoing cortical neurodegeneration throughout disease progression. Given all of that, GI might offer a more sensitive and objective measurement for studying the cognitive decline in T2DM patients. It is somewhat surprising that only one study, to date, has explored the gyrification changes in patients with T2DM (Crisostomo et al., 2021). However, whether and how gyrification is associated with cognition in T2DM patients with MCI remains unclear.

Most T2DM-associated neuroimaging studies were performed by single-modal MRI, but the heterogeneity of those studies makes it difficult to draw firm conclusions between T2DM-related MCI and neuroimaging markers. A meta-analysis suggested multimodal MRI method would be a powerful tool to overcome the inconsistencies across different studies (Rosenberg et al., 2018). Previous studies have shown that the structure of the human brain is closely related to its function, and multimodal neuroimaging approaches are more effective in exploring structural and functional changes in the brain simultaneously (Biessels and Reijmer, 2014; Rosenberg et al., 2018). Thus, based on the combination of GI and RSFC analysis, our work may provide insight into the underlying pathogenic mechanisms in T2DM patients with MCI. In the current study, we hypothesized the following: (1) Altered local gyrification and RSFC could be observed in the T2DM-MCI group when compared with the T2DM-NC group and HC group. (2) The altered GI would be related to cognitive ability and clinical variables in patients with T2DM-MCI.

## Materials and methods

### Subjects

This study was approved by the ethics committee of the Affiliated Drum Tower Hospital of Nanjing University Medical School and conducted between January 2017 and June 2020. All patients with T2DM were recruited at the outpatient and inpatient departments of the Affiliated Drum Tower Hospital of Nanjing University Medical School. T2DM participants were divided into two groups: the T2DM-MCI group (*n* = 126) and the T2DM-NC group (*n* = 154). T2DM diagnosis was based on the latest American Diabetes Association criteria (Harreiter and Roden, 2019). MCI was diagnosed based on the criteria proposed by the National Institute on Aging–Alzheimer's Association (NIA-AA) work groups: (1) a reported decline in cognitive function (self/informant/clinician report); (2) objective evidence of impairment in one or more cognitive domains, which were assessed by the Montreal Cognitive Assessment (MoCA) in our study; (3) preservation of normal activities of daily living (ADL), measured by ADL questionnaire; (4) absence of dementia (Albert et al., 2011). Participants without T2DM and cognitive impairment were defined as healthy controls, and they were recruited from a free clinic or the community during the same time (between January 2017 and June 2020). All participants have provided written informed consent. All participants were right-handed and were aged between 45 and 80 years. The exclusion criteria for all subjects were as follows: (1) age <45 years. (2) Diabetes other than T2DM (e.g., prediabetes and T1DM). (3) Acute metabolic complications such as diabetic ketoacidosis. (4) The major macrovascular and microvascular complications such as stroke, cardiovascular disease, renal insufficiency, and diabetic neuropathy. (5) History of other neurological diseases that may affect cognition (e.g., Alzheimer's disease and epilepsy). (6) Contraindications for MRI and/or inability to undergo cognitive tests or failure to complete the assessment.

### Demographic and clinical data

Participants' demographic data (i.e., age, gender, education, height, and weight), lifestyle factors (i.e., drinking and smoking), body mass index (BMI) ([weight in kg]/[height in m]^2^), and T2DM duration were obtained from questionnaires and medical records. Hypertension was defined as systolic blood pressure (SBP) ≥140 mmHg, diastolic blood pressure (DBP) ≥90 mmHg, or a history of hypertension or indicated by a record of anti-hypertensive therapy. Smokers were defined as those who smoked at least once (one or more cigarettes at a time) in the preceding year, and drinkers were defined as those who drank alcohol for more than 2 days (one or more glasses at a time) a week. The levels of glycosylated hemoglobin (HbA1c), fasting plasma glucose (FPG), total cholesterol (TC), triglyceride (TG), low-density lipoprotein (LDL), and high-density lipoprotein (HDL) were measured in a standard laboratory within 1 day of the MRI scans.

### Cognitive tests

Cognitive tests for all participants were completed by a professional neuropsychologist within 1 day of the MRI scans. As used in previous studies (Chen et al., 2019; Huang et al., 2020), the Mini-Mental State Examination (MMSE) and Montreal Cognitive Assessment (MoCA) were conducted to evaluate global cognition. In addition, the five cognitive domains and each raw test were (1) episodic memory: the auditory verbal learning test-long time delay recall (AVLT-LTDR) test and the visual reproduction-delay recall (VR-DR) test, (2) visual-spatial ability: the VR-copy (VRC) test and the clock drawing test (CDT), (3) executive function: the trail making test (TMT)B-A and the Stroop color and word test (SCWT)C-B, (4) language ability: the category verbal fluency test (CVFT) and the Boston naming test (BNT), and (5) processing speed: the TMTA and the SCWTB. Each raw test score was standardized into Z-scores and averaged to obtain one composite Z-score, which represents the overall performance of global cognition and five cognitive domains. Z-score could be computed as follows:


$$\begin{array}{l} Z\;=\;\frac{\text{x}-\bar{\text{x}}}{\text{S}} \end{array}$$

where x is the raw scores, $\bar{x}$ is the mean of raw scores, and S is the standard deviation. It is noteworthy that the results from the SCWT and TMT, which represent time, were inversely transformed to maintain consistency.

### Magnetic resonance imaging acquisition

All MRI scans were acquired using a 3.0-T scanner (Philips, the Netherlands) equipped with a 32-channel head coil. The examination protocol included a three-dimensional T1-weighted sequence (3D T1) [repetition time (TR) = 9.8 ms; echo time (TE) = 4.6 ms; flip angle (FA) = 8°; number of slices = 192; acquisition matrix = 256 × 256; thickness = 1.0 mm; and FOV= 250 x 250 mm^2^]; a three-dimensional fluid-attenuated inversion recovery (FLAIR) sequence [TR = 4,500 ms, TE = 333 ms, number of slices = 200, voxel size = 0.95 × 0.95 × 0.95 mm^3^, and acquisition matrix = 270 × 260]; and a BOLD sequence, including 230 volume (TR = 2,000 ms, TE = 30 ms, FA = 90°, number of slices = 35, acquisition matrix = 64 × 64, FOV = 230 × 230 mm^2^, and thickness = 4.0 mm). Participants were asked to keep their eyes closed and not to fall asleep or think about anything in particular during the scan.

### GI analysis

The CAT12 toolbox (http://dbm.neuro.uni-jena.de/cat/) based on SPM12 (http://www.fil.ion.ucl.ac. uk/spm/software/spm12/) software was used to process and analyze all 3D T1-weighted images. CAT12 sets a processing pipeline for SBM, which allows the estimation of the gyrification index based on the absolute mean curvature approach (Luders et al., 2005). This tool has been previously used and validated in cortical studies in many neurological and psychiatric diseases. We used default settings in the process and analysis steps, and the east Asian brains ICBM space template was used for preprocessing steps. Processing included two procedures for quality assurance. First, all images were visually checked for artifacts (prior to preprocessing); second, all images underwent statistical quality control and inter-subject homogeneity after segmentation. The latter procedure again included a visual inspection for potential artifacts that were newly introduced. GI maps were calculated based on the absolute mean curvature approach (Dahnke et al., 2013). Extraction of the cortical surface contributed to the construction of a mesh of the central surface (i.e., the surface between the gray matter/white matter border and the gray matter/CSF boundary). Then, we calculated the local absolute mean curvature of the central surface by averaging the mean curvature values from each vertex point within 3 mm from a given point. Finally, 15 mm full width at half maximum (FWHM) was applied to smooth the GI maps.

### RSFC analysis

#### Preprocessing

The original functional image data were analyzed using DPABI (for Data Processing and Analysis of Brain Imaging, http://rfmri.org/dpabi) (Yan et al., 2016) and SPM12. Preprocessing included the following steps: (1) removal of the first 10 volumes for magnetization equilibrium, (2) slice-timing correction was performed to correct the acquisition time delay between slices, (3) head motion correction, (4) realignment, (5) spatial normalization of the realigned images to standard MNI space, (6) sampling of the normalized images to 3 × 3 × 3 mm^3^ and smoothing with a 6-mm full width at half maximum (FWHM) isotropic Gaussian kernel, (7) temporal bandpass filtering (0.01–0.08 Hz), and (8) elimination of nuisance variables (head motion parameters and WM signals, CSF signals, and global signals).

#### Seed-based RSFC analysis

Referencing previous literature, clusters showing significant between-group differences in GI analysis were extracted as seed regions (Wang et al., 2018). First, the peak coordinate for each significant cluster obtained from GI analysis was selected to create 5-mm radius regions of interest (ROIs). Then, we calculated Pearson's correlation between the mean time course of each seed and the time series of each of the remaining voxels throughout the whole brain to generate the FC map. Next, the FC maps were further converted to z-values using Fisher's z transformation for standardization. Finally, functionality images for each subject were ultimately obtained.

### CSVD imaging markers

Lacune was defined as a round or ovoid cavity (generally they are 3–15 mm in diameter). On FLAIR images, lacunes generally have a central CSF-like hypointensity with a surrounding rim of hyperintensity, but the rim is not always present (Wardlaw et al., 2013). In addition, the volumes of white matter hyperintensities (WMH) were automatically calculated by the Wisconsin WMH Segmentation Toolbox (W2MHS, https://sourceforge.net/projects/w2mhs) on 3D T1 and FLAIR images (Ithapu et al., 2014). WMH results included three items: total white matter hyperintensity (TWMH), periventricular white matter hyperintensity (PVWMH), and deep white matter hyperintensity (DWMH).

### Statistical analysis

Patients' demographic, clinical, and cognitive data (e.g., age, sex, education, BMI, HbA1c, LDL, global cognition, and five cognitive domains) were analyzed with SPSS 22. First, the Shapiro–Wilk test was used to determine the normality of the continuous data. When data were normally distributed, a one-way analysis of variance (ANOVA) or independent sample *t*-test was conducted to detect differences between the three groups. If the assumption of normality was not met, a nonparametric Kruskal–Wallis H-test was performed to compare between-group differences. Additionally, a chi-square test was conducted to estimate group differences in dichotomous variables (e.g., gender, hypertension, smoking, and drinking). The statistical significance level was set at *p* < 0.05. The GI/RSFC differences among the three groups were detected by ANOVA in the CAT12/SPM12 statistical module [familywise error (FWE) correction, p < 0.05]. Variables that were significant in univariate analysis were entered into GI and RSFC analysis as covariates (education, FPG, and PVWMH). In addition, some variables (age and gender) were known to be associated with cognitive function, so they were also taken as covariates too. When there was a significant between-group effect, we performed *post-hoc* pairwise comparisons (Bonferroni corrected) to determine differences between every pair of groups. Pearson's correlation was conducted to explore the correlation among GI, clinical data, and cognitive scores in the T2DM-MCI group and T2DM-NC group, and statistical significance was set at *P* < 0.05. In addition, mediation analysis was performed to examine the mediating effect of RSFC on the association between GI and cognition (global cognition and episodic memory), controlling for age, gender, education, FPG, and PVWMH. IBM SPSS PROCESS (v3.2, model 4) was used for mediation analysis. The bootstrapping method (based on 5,000 resamples) was conducted to estimate the corresponding 95% confidence interval (CI). The mediation effect was considered to be significant if the bootstrap 95% CI of the β coefficient did not include zero.

## Result

### Demographic and clinical data

Demographic and clinical data for participants of the three groups are summarized in Table 1. Participants in the T2DM-MCI group had higher levels of HbA1c (*P* < 0.001) and lower levels of education (*P* < 0.001) than participants in the two other groups. Participants in the T2DM-MCI group and T2DM-NC group had higher FPG levels than participants in the HC group (*P* < 0.001), but there was no significant difference in FPG levels between the two T2DM groups. The T2DM-MCI group had a larger volume of PVWMH than the HC group. Participants in the T2DM-MCI group performed worse than participants in the other two groups on assessments of global cognition (*P* < 0.001), episodic memory (*P* < 0.001), visual-spatial ability (*P* < 0.001), and language ability (*P* < 0.001). In addition, there was no significant difference in these indices (HbA1c, education, global cognitive function, working memory, visual-spatial ability, and language ability) between participants in the T2DM-NC and HC groups. No significant differences were observed in age, gender, BMI, TC, TG, HDL, LDL, hypertension, smoking, drinking, TIV, TWMH volume, DWMH volume, executive function, or processing speed among the three groups. The T2DM duration in the T2DM-MCI group was significantly longer than that in the T2DM-NC group (*P* = 0.034). In addition, the raw scores of each cognitive test for participants of the three groups are shown in Supplementary Table S1.

**Table 1:** Demographic, clinical, and cognitive data for participants of the three groups.

	**HC**	**T2DM-NC**	**T2DM-MCI**	**t/F/χ2 value**	***P*-value**
	**(*n* = 167)**	**(*n* = 154)**	**(*n* = 126)**		
Age (years)	61.79 (15)	62.14 (14)	61.66 (12)	0.367	0.873
Gender (male, %)	84 (50.30%)	93 (60.39%)	59 (46.83%)	5.850	0.054
Education (years)	12 (6)	9 (4)	9 (3)	15.642	<0.001^a, b^
T2DM duration (years)	-	6.41 ± 5.03	7.93 ± 6.93	176.214	0.034^b^
BMI (kg/m^2^)	24.68 ± 4.37	25.09 ± 3.18	24.04 ± 2.74	1.908	0.151
HbA1c (%)	5.3 (0.9)	6.5 (0.9)	6.6 (1.6)	217.188	<0.001^a, b^
FPG (mmol/L)	4.98 ± 0.68	6.04 ± 1.74	6.27 ± 2.14	29.443	<0.001^a, c^
TC (mmol/L)	4.48 ± 1.14	4.29 ± 0.97	4.02 ± 1.74	2.149	0.183
TG (mmol/L)	1.41 ± 0.73	1.43 ± 0.78	1.47 ± 1.14	0.313	0.852
HDL (mmol/L)	1.31 ± 0.45	1.22 ± 0.36	1.32 ± 0.44	1.748	0.276
LDL (mmol/L)	2.60 ± 0.94	2.48 ± 0.78	2.23 ± 0.65	2.175	0.094
Hypertension (n, %)	97 (58.08%)	98 (63.64%)	84 (66.67%)	1.844	0.398
Smoking (n, %)	42 (25.15%)	51 (33.11%)	34 (26.98%)	4.533	0.399
Drinking (n, %)	50 (29.94%)	47 (30.52%)	27 (21.43%)	3.599	0.165
TIV (cm^3^)	1431.8 ± 211.6	1466 ± 204.1	1434 ± 196.42	1.484	0.347
TWMH volume (ml)	3.10 ± 2.13	3.37 ± 2.25	3.73 ± 2.51	2.738	0.066
PVWMH volume (ml)	0.77 ± 0.58	0.86 ± 0.61	0.97 ± 0.68	3.594	0.028^a^
DWMH volume (ml)	2.33 ± 1.55	2.53 ± 1.69	2.76 ± 1.84	2.389	0.093
Lacunes (number)	1.17 ± 1.25	1.37 ± 1.69	1.29 ± 1.63	0.748	0.474
Z-Global cognition	0.44 ± 0.49	0.29 ± 0.55	−0.71 ± 1.02	108.872	<0.001^a, b^
Z-Executive function	0.57 ± 121.36	5.0 ± 56.95	6.47 ± 63.53	0.181	0.835
Z-Processing speed	−4.20 ± 17.13	−3.95 ± 19.60	−2.58 ± 18.61	0.307	0.736
Z-Episodic memory	0.27 ± 0.73	0.13 ± 0.70	−0.41 ± 0.79	32.602	<0.001^a, b^
Z-Language function	0.23 ± 0.61	0.17 ± 0.70	−0.41 ± 0.82	34.481	<0.001^a, b^
Z-Visual-spatial ability	0.14 ± 0.62	0.11 ± 0.53	−0.27 ± 1.09	12.554	<0.001^a, b^

*P* < 0.05 had statistical significance.

^a^Compare HC group to T2DM-MCI group,

^b^Compare T2DM-NC group to T2DM-MCI group,

^c^Compare HC group to T2DM-NC group.

BMI, body mass index; FPG, fasting plasma glucose; TC, total cholesterol; TG, triglyceride; HDL, high-density lipoprotein; LDL, low-density lipoprotein; TIV, estimated total intracranial volume; TWMH, total white matter hyperintensity; DWMH, deep white matter hyperintensity; PVWMH, periventricular white matter hyperintensity.

### GI analysis and seed-based RSFC analysis

**Figure 1:**
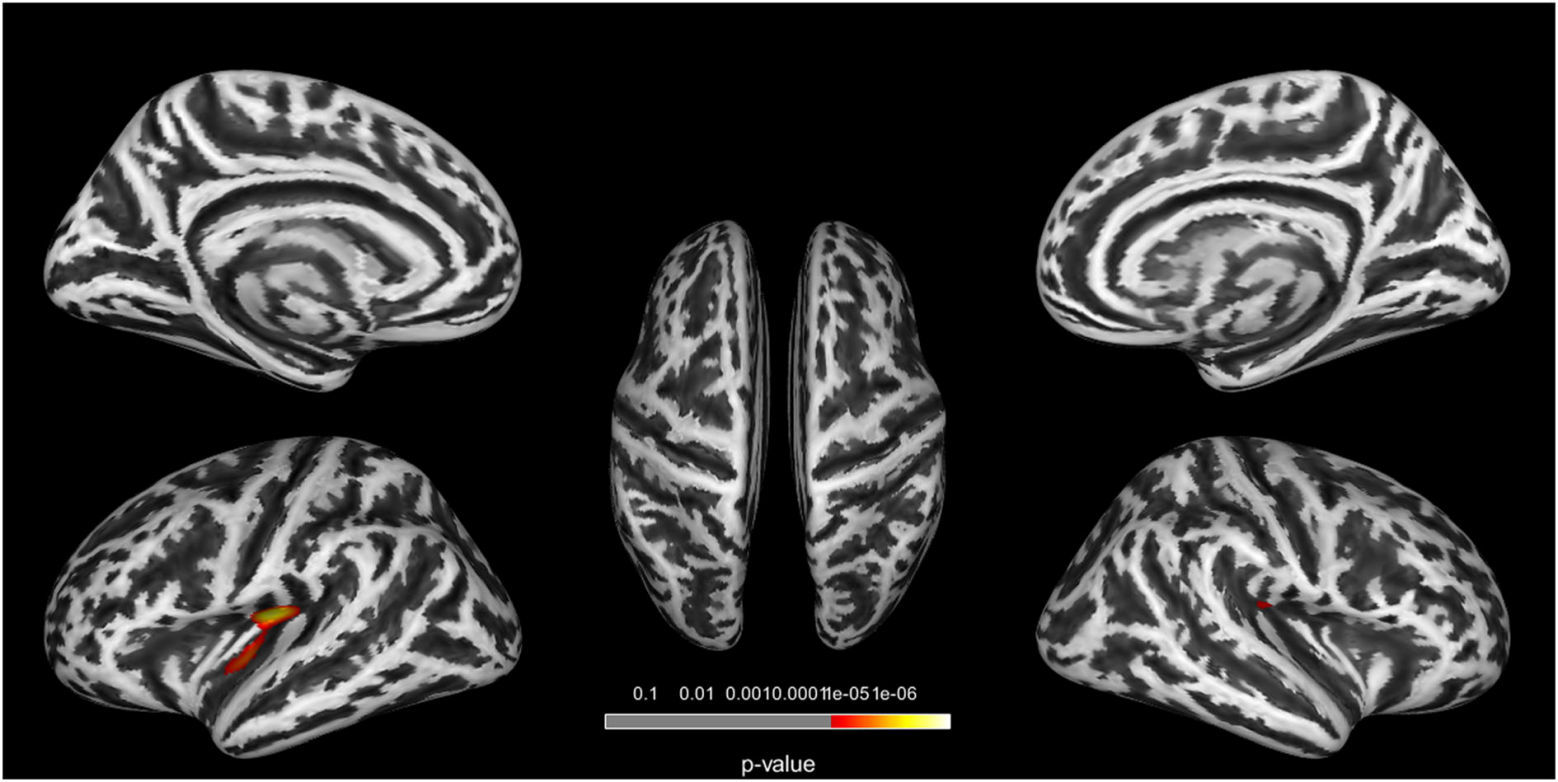
Clusters with significant group differences in GI analysis. Corrected for age, gender, education, FPG, and PWMH (FWE corrected, *p* < 0.05).

In the GI analysis among three groups, two clusters located in the bilateral insula revealed a group effect (Figure 1). Cluster 1 is located in the left insula (cluster size = 239 vertices, peak MNI coordinates: x = −29, y = −19, z = 29, and cluster-level *p*<0.001). Cluster 2 is located in the right insula (cluster size = 114 vertices, peak MNI coordinates: x = 38, y = −21, z = 32, and cluster-level *p* = 0.023) (Table 2). *Post-hoc* analyses in three clusters showed decreased GI in the T2DM-MCI patients compared to the T2DM-NC group and HC group. We did not observe significant differences between participants in the T2DM-NC and HC groups (Supplementary Figure S1).

**Table 2:** Clusters with significant group differences in GI analysis.

**Cluster**	**Brain regions**	**Cluster size (number of vertex)**	**Peak MNI coordinates**			***p*-value (cluster-level)**	***p*-value (peak-level)**
			**x**	**y**	**Z**		
1	Left insular cortex	239	−29	−19	29	<0.001	0.001
2	Right insula cortex	114	38	−21	32	0.023	0.027

Reported clusters survived FWE correction at *P* < 0.05, using age, education, FPG, and PWMH as covariates.

MNI, Montreal Neurological Institute.

### Seed-based RSFC analysis

**Figure 2:**
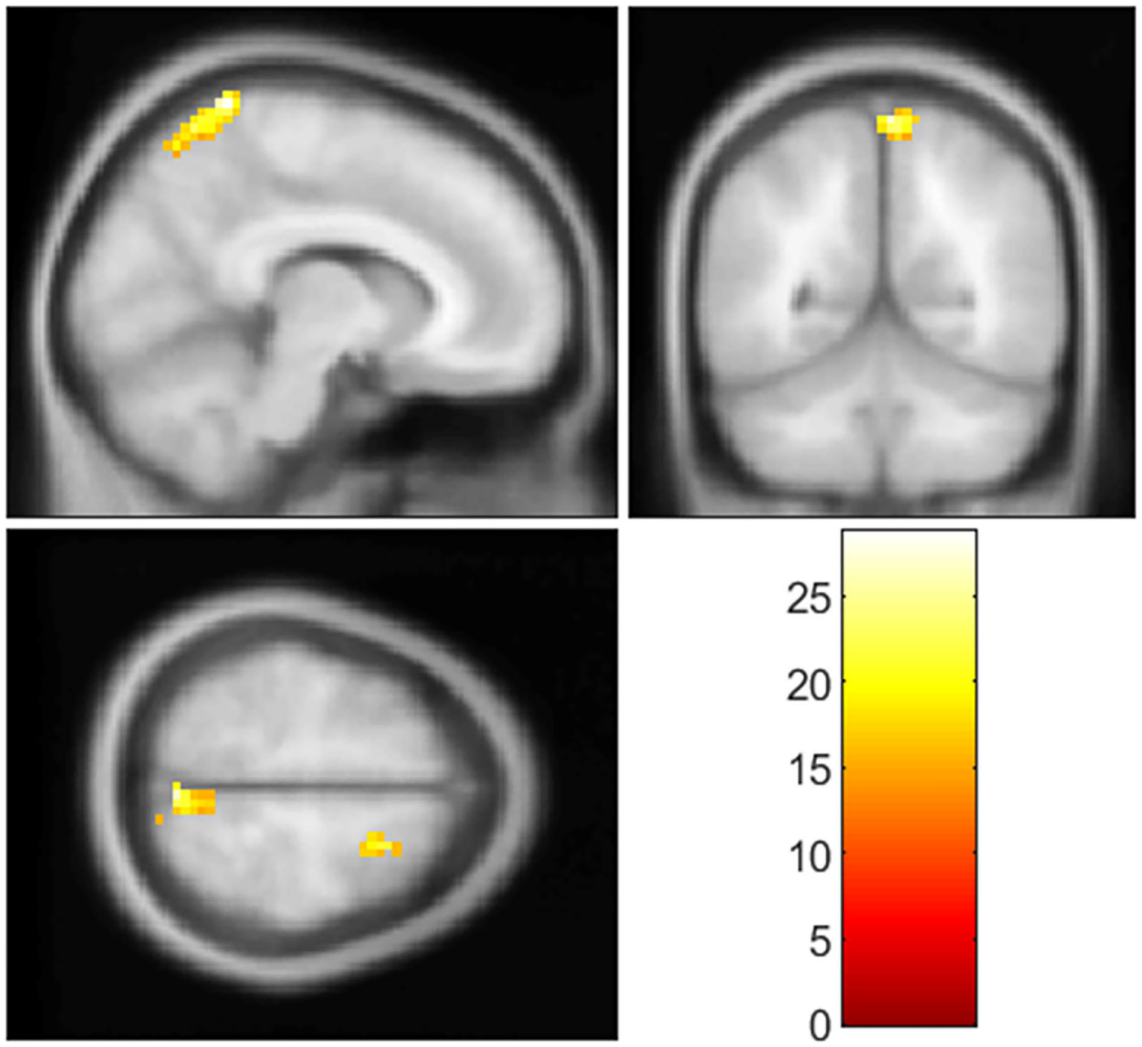
Clusters with significant group differences in RSFC analysis. Corrected for age, gender, education, FPG, PWMH, and TIV (FWE corrected, *p* < 0.05).

As mentioned above, clusters showing significant between-group differences in GI analysis were extracted as seed regions. In the RSFC analysis among three groups, decreased RSFC was found between left insular and right precuneus (cluster size = 116 voxels, peak MNI coordinates: x = 9, y = −48, z = 75, and cluster-level *p*<0.001), and right SFG (cluster size = 188 voxels, peak MNI coordinates: x = 21, y = 9, z = 60, and cluster-level *p*<0.001) when used cluster 1 as seed region (Figure 2, Table 3). *Post-hoc* analysis showed (1) RSFC between the left insular and right SFG, and right precuneus in the T2DM-MCI group was significantly decreased when compared to the T2DM-NC group and HC group, and (2) no significant differences in RSFC were observed between T2DM-NC group and HC group (Supplementary Figure S2). Moreover, no significant differences in RSFC were observed among the three groups when we used cluster 2 as the seed region.

**Table 3:** Clusters with significant group differences in RSFC analysis.

**Cluster**	**Brain regions**	**Cluster size (number of voxel)**	**Peak MNI coordinates**	***p*-value (cluster-level)**	***p*-value (peak-level)**
			**x**	**y**	**z**		
A	Right SFG	188	21	9	60	<0.001	<0.001
B	Right Precuneus	116	9	−48	75	<0.001	<0.001

Reported clusters survived FWE correction at *P* < 0.05, using age, education, FPG, PWMH, and TIV as covariates.

MNI, Montreal Neurological Institute; SPG, superior parietal gyrus.

### Correlational analysis and mediation analysis

**Figure 3:**
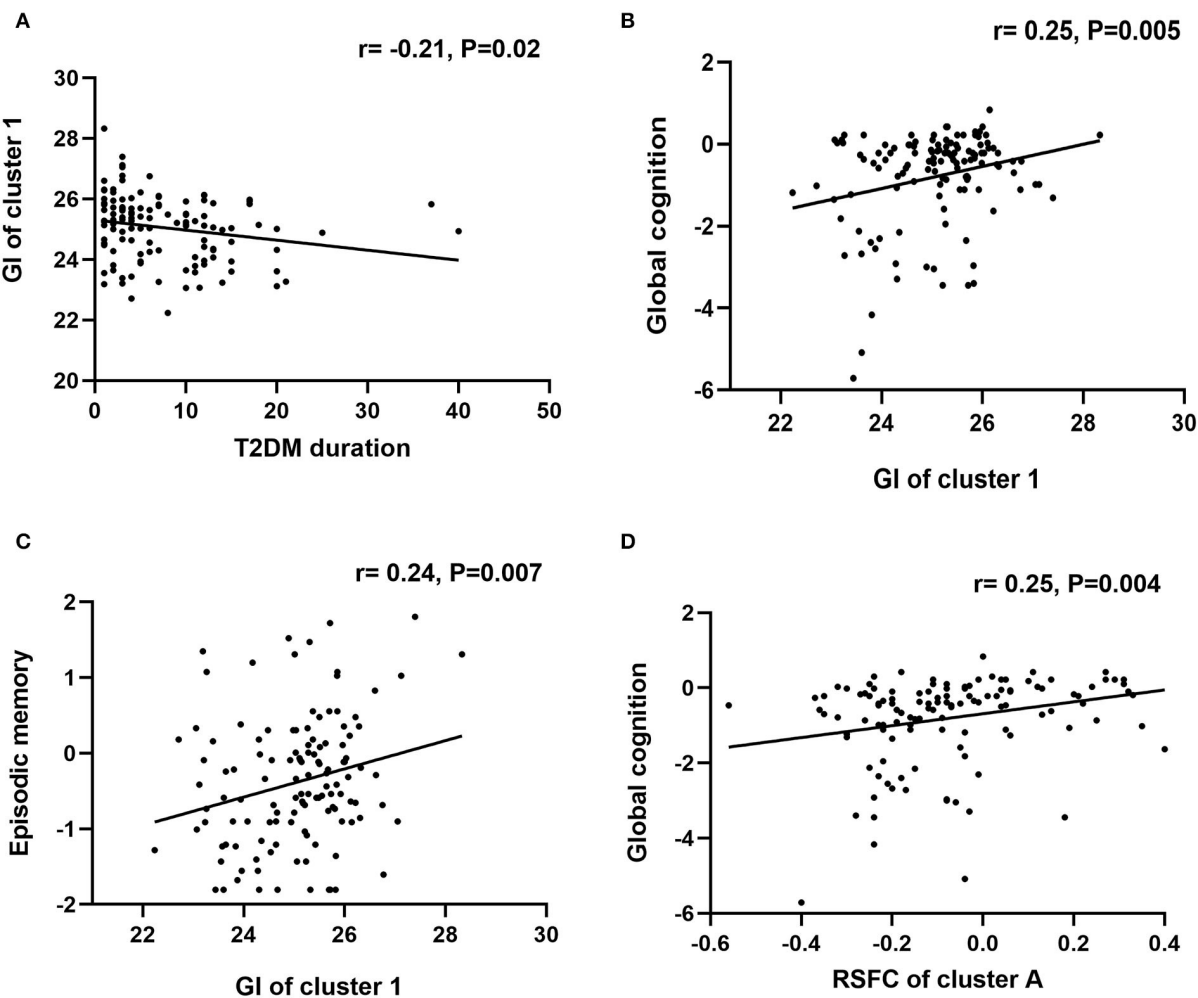
Correlational analysis in the T2DM-MCI group. **(A)** Correlation analysis between T2DM duration and GI of cluster 1. **(B)** Correlation analysis between GI of cluster 1 and global cognition. **(C)** Correlation analysis between GI of cluster 1 and episodic memory. **(D)** Correlation analysis between RSFC of cluster A and global cognition.

**Figure 4:**
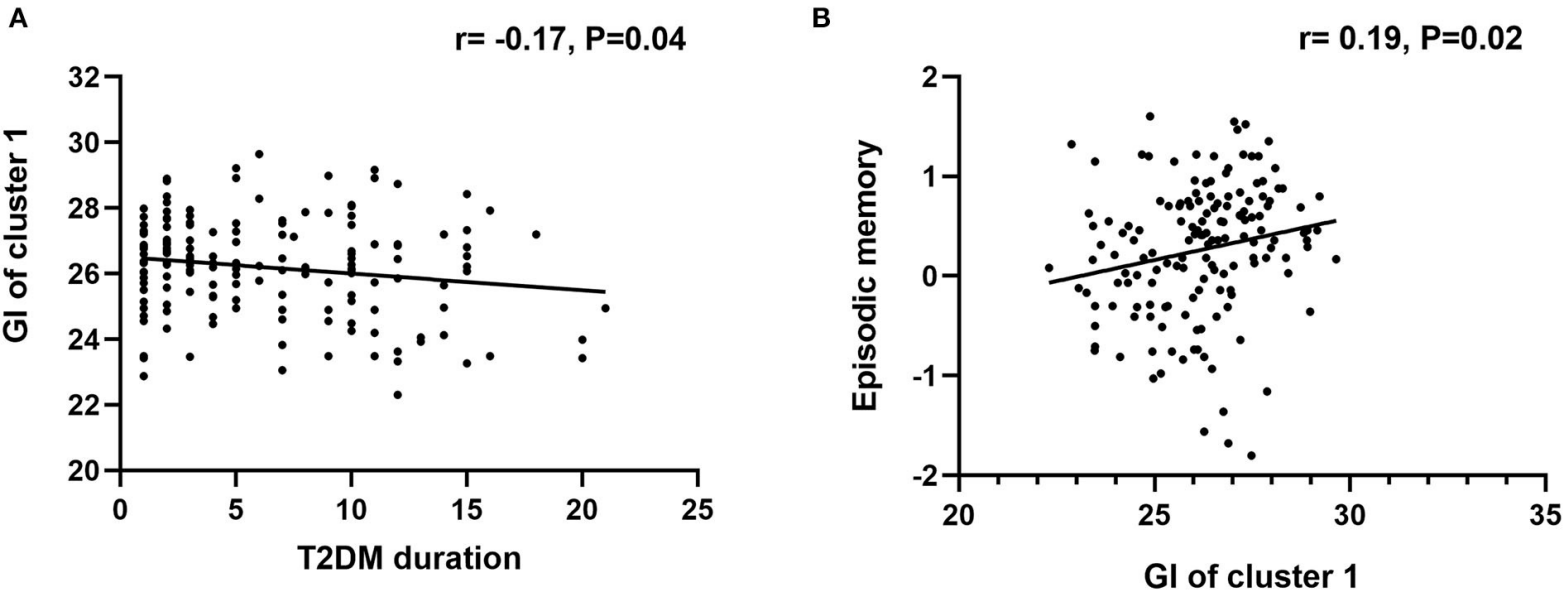
Correlational analysis in the T2DM-NC group. **(A)** Correlation analysis between T2DM duration and GI of cluster 1. **(B)** Correlation analysis between GI of cluster 1 and episodic memory.

Correlation analyses were conducted in the T2DM-MCI group. Between the two clusters showing significant between-group differences in GI analysis, only the GI of cluster 1 was significantly correlated with the duration of T2DM (r = −0.21, *P* = 0.02). The GI of cluster 1 was positively correlated with global cognition (r =0.25, *P* = 0.005), and episodic memory (r = 0.24, *P* = 0.007). In addition, the RSFC between the left insula and right SFG was positively correlated with global cognition (r =0.25, *P* = 0.004) (Figure 3). Moreover, the T2DM duration was not correlated with any cognitive domains. No significant correlations between GI and the level of HbA1c were observed, and the GI of cluster 2 was not correlated with any cognitive domains. We also conducted correlation analysis in the T2DM-NC group, and the results showed that 1) GI of cluster 1 was significantly correlated with the duration of T2DM (r = −0.17, *P* = 0.04), and 2) GI of cluster 1 was positively correlated with episodic memory (r = 0.19, *P* = 0.02) (Figure 4).

**Figure 5:**
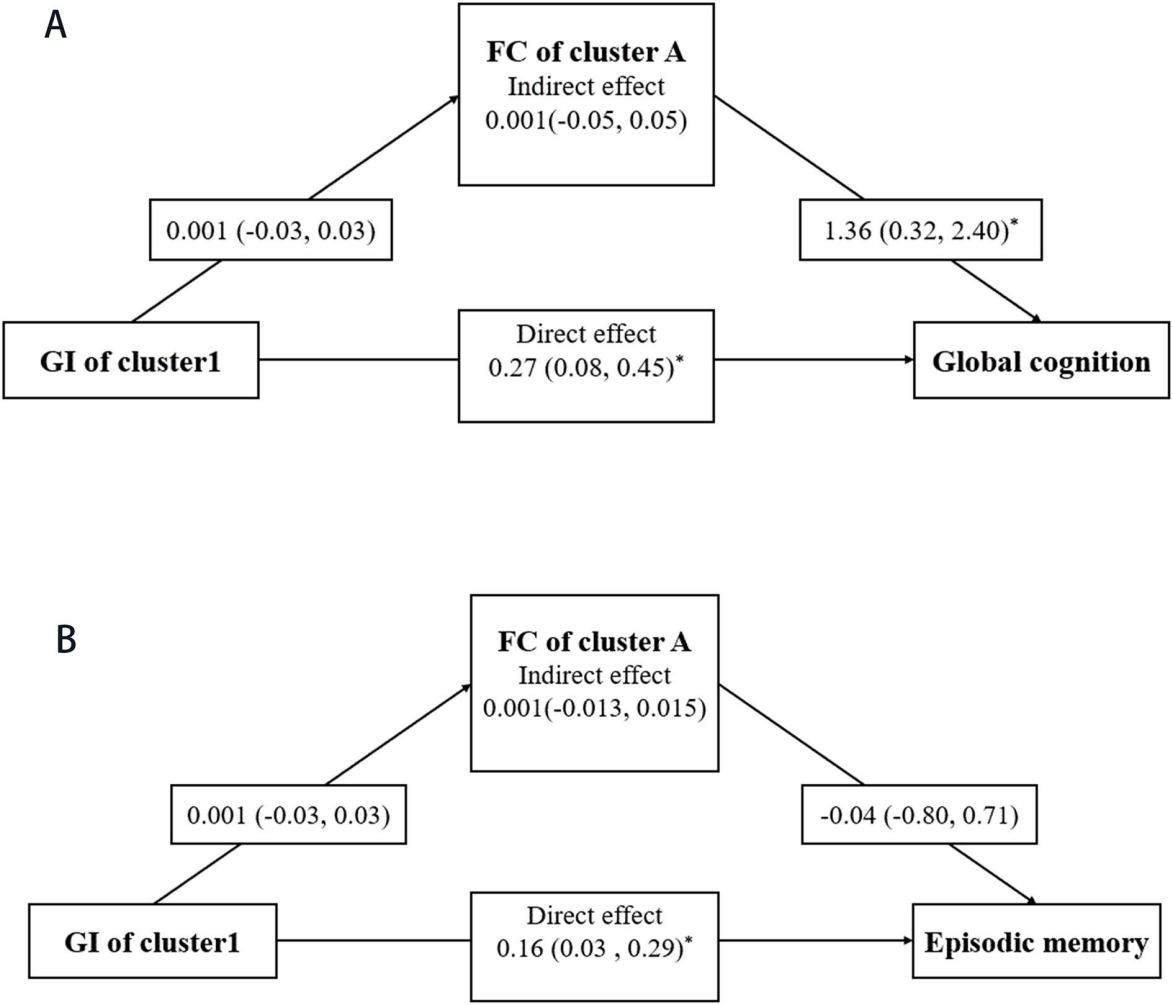
Mediation analysis. Mediating effects of RSFC in the associations of GI with global cognition **(A)** and episodic memory **(B)**. Mediation models were adjusted for age, gender, education, FPG, and PWMH. For each connection, the β coefficient and corresponding 95% confidence interval (CI) are shown. * represents the significant pathway (*p* < 0.05).

The mediation effects of RSFC on the associations of GI with cognitive function were assessed in the T2DM-MCI group. After controlling for age, gender, education, FPG, and PVWMH, the mediation effects of RSFC on the association between GI and global cognition and episodic memory were not statistically significant (*p* > 0.05) (Figure 5).

## Discussion

To our knowledge, this is the first study to combine GI and RSFC approaches to reveal the neural basis of T2DM-related MCI. First, compared with T2DM-NC and HC groups, participants in the T2DM-MCI group showed significantly decreased GI in the bilateral insular cortex. Second, using the clusters derived from the GI analysis as seed regions, decreased RSFC was found between left insular and right SFG and right precuneus in the T2DM-MCI group. Third, the aberrant GI of the left insula was negatively correlated with T2DM duration, and it was positively correlated with global cognition and episodic memory. Fourth, the RSFC between the left insula and right SFG was positively correlated with episodic memory. Our findings provide valuable information that may improve the present understanding of T2DM-related neuroimaging alterations in the central neural system.

### Putative mechanisms of aberrant GI and RSFC

There are several possible explanations for the decreased gyrification in patients with T2DM-MCI. First, the “tension-based” theory proposed that white matter connectivities (both cortico-cortical and cortico-subcortical) produce local tension, which brings interconnected brain regions together and creates folding (Van Essen, 1997). Altered cortico-cortical and cortico-subcortical networks have been reported frequently in patients with T2DM in previous studies (Hsu et al., 2012; Zhang et al., 2014). It is reported that disrupted fiber pathways result in gyrification changes in the monkey brain (Goldman and Galkin, 1978). Second, the “differential tangential expansion” theory postulated that diverse rates of growth between cortical layers led to buckling of the cortical surface and then resulted in the folding of the cortex (Richman et al., 1975). Although no direct evidence of diverse cortical layer growth rates in T2DM has been provided, many studies have reported reductions in gray matter volume and thickness in T2DM patients (Rosenberg et al., 2018). We speculate that gray matter atrophy underlies changes in gyrification. Third, environmental factors like diet were also found to have an effect on cortical gyrification, probably due to abnormalities in cell death that normally occurs during neural pruning (Gautam et al., 2015). In addition, we did not observe altered GI in other brain regions except the insula. T2DM may cause damage in specific brain regions, and the insula may be one vulnerable target. Therefore, compared with other brain regions, altered GI might be more easily observed in the insula. A 5-year longitudinal study indicated that neurovascular coupling of the insula decreased more severely than in other brain regions in patients with T2DM (Damer and Creutz, 1994). A T2DM-associated study showed increased gyrification was found in the temporal lobes, posterior lobe, parietal lobe, and occipital lobe, and decreased gyrification was in the temporal lobe (Crisostomo et al., 2021). This study reported clusters different from our study, revealing that other brain regions may be affected by T2DM. However, the aforementioned study only included two groups of participants (T2DM group vs. control group), and the sample size was relatively small. Longitudinal studies are needed in the future to confirm our current results.

Current evidence has shown that disorders of RSFC could be detected frequently in various resting-state networks in T2DM patients, such as the default mode network (DMN), salience network (SN), dorsal attention network (DAN), and frontal-parietal control network (FPCN) (Macpherson et al., 2017; Chau et al., 2022; Meng et al., 2022). The insula is the core region of SN, and SFG and precuneus are frequently reported regions of DMN (Cavanna and Trimble, 2006; Li et al., 2013), thus, it is reasonable that the decreased RSFC between the insula and SFG and precuneus could be detected in patients with T2DM. Cerebrovascular changes, BIR, cerebrospinal fluid (CBF), and inflammation may serve as key risk factors for decreased RSFC in patients with T2DM (Cherbuin and Walsh, 2019; Xue et al., 2019).

It is worth noting that differences in the GI/RSFC were not significant between participants in the HC and T2DM-NC groups, suggesting that significant changes in the GI/RSFC in those brain regions may occur during the later stages of the T2DM. Another study on PD also suggested the accelerated decrease of gyrification becomes prominent in later stages (Sterling et al., 2016), which is similar to our results.

### Correlation analysis and mediation analysis

Type 2 diabetes mellitus duration and glycemic control are widely measured to reflect the severity of the disease. We found that the GI of the left insula was negatively correlated with the T2DM duration. However, there was no significant correlation between GI and HbA1c. Current studies have confirmed a significant correlation between T2DM duration and aberrant brain structure (gray matter thickness, gray matter volume, mean diffusivity, etc.) (Rosenberg et al., 2018). Chronic hyperglycemia (a long course of T2DM) may trigger a complex interplay between biochemical and systemic factors, leading to alterations in the GI in the cortex. HbA1c was used as a standard measure in clinical practice to monitor glycemic control. Some studies reported that the relationship between HbA1c and cognitive performance was either absent or weak (Cukierman-Yaffe et al., 2009). HbA1c only reflected glycemic control in the most recent course (8–12 weeks), which may not accurately predict the damage to the brain structure during a longer course of T2DM.

Our study showed that the GI of the insula cortex correlated with cognition in the T2DM-MCI group. Similarly, recent studies also reported that increasing gyrification of the bilateral insula was associated with higher levels of global cognition and memory function in the elderly population (Kinno et al., 2019; Lamballais et al., 2020). The insula has been recently proposed to serve as a “hub” for cognitive and affective integration (Critchley, 2005). Indeed, as an important brain region, the bilateral insular cortex has been shown to be involved in cognitive ability in many studies. A study suggested that higher gyrification shortens the distance between various white matter tracts in adjacent brain regions, which would be a prominent way to increase signaling speed and cortical communication, thus aiding in better cognition (Green et al., 2018). In addition, we also found that the GI of the insula cortex was correlated with cognition in the T2DM-NC group. It is suggested that the GI of the insula is closely related to the cognition of patients with T2DM, whether patients with T2DM have MCI or not. Combined with the correlation analysis in the T2DM-MCI group, we speculated that the accelerated decrease of GI occurs shortly after T2DM diagnosis and becomes prominent in later stages.

Our study further found decreased RSFC between the left insula and right SFG and precuneus in the T2DM-MCI group. In particular, the insular cortex has been demonstrated to play a crucial role in coordinating interactions between several neurocognitive networks that maintain normal cognitive function (Uddin, 2015). Disrupted functional connectivity of the insular cortex may contribute to the pathogenesis and development of cognitive impairment in patients with T2DM (Zhang et al., 2021). The SFG and precuneus are frequently reported regions of the DMN (Cavanna and Trimble, 2006; Li et al., 2013). As the DMN participates in the process of adaptive cognitive control and multiple advanced cognitive tasks, it has been verified by many previous studies to be implicated in deficits of various cognitive domains.

The mediation effects of RSFC in the brain structure–cognition association are occasionally reported in the previous literature (Hanly et al., 2022; Yang et al., 2022). Although it is biologically plausible to hypothesize that abnormal GI might be linked to cognitive function *via* affecting RSFC, our study did not observe a significant mediation effect. This suggests that GI and RSFC might alter in a concurrent process, which deserves further investigation.

### Multimodal MRI approach

The multimodal MRI approach could verify the true nature and etiology of imaging abnormalities in T2DM patients more effectively (Biessels and Reijmer, 2014). A few studies used a multimodal neuroimaging approach, but different modal images were processed and discussed separately, leading to a lack of interconnection in the results. In the current study, GI and RSFC analyses were considered two interrelated analytical methods, and the clusters showing group effects in GI analysis were then used as seed regions to conduct RSFC analysis. The multimodal imaging approach would be a promising tool to develop a body of understanding in the area of T2DM.

## Limitation

There are some limitations to our study. First, our cross-sectional and correlational design limited our ability to make firm causal inferences about cognitive ability, clinical variables, and the observed imaging abnormalities. In addition, the sample size of our study is relatively small, and large-scale and multiple-center longitudinal studies are needed in the future to draw more accurate and convincing conclusions. Second, some studies employed different methodologies for measuring GI (Schaer et al., 2008), while our current study applied the absolute mean curvature approach, which has been performed in a range of applications and thus might not be completely comparable with the findings of other studies. Third, cerebrospinal fluid was used to define the etiologic subtypes of MCI strictly. However, cerebrospinal fluid was not available for many participants. Patients in this study were recruited based on clinical criteria, so it is difficult to completely rule out the possibility of Alzheimer's disease which is related to the present result. This is the general limitation of research in this field (Gao et al., 2019; Zhang et al., 2019). Fourth, more subgroups (MCI-Non-T2DM and T2DM-dementia) should be involved in a future study to explore more meaningful results in patients with T2DM. Despite these limitations, we believe our results exhibit reliable information that is necessary for understanding the underlying neuropathological mechanisms of T2DM-related MCI.

## Conclusion

Using a multimodal approach of GI and RSFC analysis, this study investigated the roles of structural and functional change patterns in the brains of patients with T2DM-MCI. Taken together, our results suggested that the GI may act as a potential biomarker to assess and to better understand the neuropathology of T2DM-related MCI.

## Data availability statement

The original contributions presented in the study are included in the article/Supplementary material, further inquiries can be directed to the corresponding authors.

## Ethics statement

The studies involving human participants were reviewed and approved by the Drum Tower Hospital Research Ethics Committee. The patients/participants provided their written informed consent to participate in this study.

## Author contributions

BZ, HZ, and YX: conceptualization and methodology. PS, XL, and HX: MRI data acquisition and thesis writing. XS, RQ, JM, and LH: cognitive tests. YC and HC: statistics and validation. All authors contributed to the article and approved the submitted version.

## Funding

This study was funded by grants from the National Natural Science Foundation of China (81630028 and 81771157), the Key Research and Development Program of Jiangsu Province of China (BE2016610), Jiangsu Province Key Medical Discipline (ZDXKA2016020), Jiangsu Province 333 Project, and Nanjing Medical Science and Technique Development Foundation.

## Conflict of interest

The authors declare that the research was conducted in the absence of any commercial or financial relationships that could be construed as a potential conflict of interest.

## Publisher's note

All claims expressed in this article are solely those of the authors and do not necessarily represent those of their affiliated organizations, or those of the publisher, the editors and the reviewers. Any product that may be evaluated in this article, or claim that may be made by its manufacturer, is not guaranteed or endorsed by the publisher.

## Abbreviations

AVLT-LTDR: the auditory verbal learning test-long time delay recall test
BMI: body mass index
BNT: the Boston naming test
BIR: brain insulin resistance
CDT: the clock drawing test
CSF: cerebrospinal fluid
CVFT: the category verbal fluency test
FPG: fasting plasma glucose
GI: gyrification index
HbA1c: glycosylated hemoglobin
HC: healthy controls
HDL: high-density lipoprotein
LDL: low-density lipoprotein
MCI: mild cognitive impairment
MFG: middle frontal gyrus
MMSE: Mini-Mental State Examination
MoCA: Montreal Cognitive Assessment
MRI: magnetic resonance imaging
ROIs: regions of interest
RSFC: resting-state functional connectivity
SBM: surface-based morphometry
SFG: superior frontal gyrus
T2DM: type 2 diabetes mellitus
TC: total cholesterol
TG: Triglyceride
VBM: voxel-based morphometry
WMH: white matter hyperintensities.

## References

[B1] Albert M. S. DeKosky S. T. Dickson D. Dubois B. Feldman H. H. Fox N. C.. (2011). The diagnosis of mild cognitive impairment due to Alzheimer's disease: recommendations from the National Institute on Aging-Alzheimer's Association workgroups on diagnostic guidelines for Alzheimer's disease. Alzheimers Dement. 7, 270–279. 10.1016/j.jalz.2011.03.008 21514249 PMC3312027

[B2] Bellary S. Kyrou I. Brown J. E. Bailey C. J. (2021). Type 2 diabetes mellitus in older adults: clinical considerations and management. Nat Rev Endocrinol. 17, 534–548. 10.1038/s41574-021-00512-2 34172940

[B3] Biessels G. J. Reijmer Y. D. (2014). Brain changes underlying cognitive dysfunction in diabetes: what can we learn from MRI? Diabetes. 63, 2244–2252. 10.2337/db14-0348 24931032

[B4] Cavanna A. E. Trimble M. R. (2006). The precuneus: a review of its functional anatomy and behavioural correlates. Brain. 129, 564–583. 10.1093/brain/awl004 16399806

[B5] Chau A. C. M. Smith A. E. Hordacre B. Kumar S. Cheung E. Y. W. Mak H. K. F.. (2022). Scoping review of resting-state brain functional alterations in type 2 diabetes. Front Neuroendocrinol. 65, 100970. 10.1016/j.yfrne.2021.100970 34922997

[B6] Chen X. Huang L. Ye Q. Yang D. Qin R. Luo C.. (2019). Disrupted functional and structural connectivity within default mode network contribute to WMH-related cognitive impairment. Neuroimage Clin. 24, 102088. 10.1016/j.nicl.2019.102088 31795048 PMC6861557

[B7] Cherbuin N. Walsh E. I. (2019). Sugar in mind: untangling a sweet and sour relationship beyond type 2 diabetes. Front Neuroendocrinol. 54, 100769. 10.1016/j.yfrne.2019.100769 31176793

[B8] Coalson T. S. Van Essen D. C. Glasser M. F. (2018). The impact of traditional neuroimaging methods on the spatial localization of cortical areas. Proc Natl Acad Sci U S A. 115, E6356–E65. 10.1073/pnas.1801582115 29925602 PMC6142239

[B9] Crisostomo J. Duarte J. V. Moreno C. Gomes L. Castelo-Branco M. (2021). A novel morphometric signature of brain alterations in type 2 diabetes: patterns of changed cortical gyrification. Eur J Neurosci. 54, 6322–6333. 10.1111/ejn.15424 34390585 PMC9291170

[B10] Critchley H. D. (2005). Neural mechanisms of autonomic, affective, and cognitive integration. J Comp Neurol. 493, 154–166. 10.1002/cne.20749 16254997

[B11] Cukierman T. Gerstein H. C. Williamson J. D. (2005). Cognitive decline and dementia in diabetes–systematic overview of prospective observational studies. Diabetologia. 48, 2460–2469. 10.1007/s00125-005-0023-4 16283246

[B12] Cukierman-Yaffe T. Gerstein H. C. Williamson J. D. Lazar R. M. Lovato L. Miller M. E.. (2009). Relationship between baseline glycemic control and cognitive function in individuals with type 2 diabetes and other cardiovascular risk factors: the action to control cardiovascular risk in diabetes-memory in diabetes (ACCORD-MIND) trial. Diabetes Care. 32, 221–226. 10.2337/dc08-1153 19171735 PMC2628683

[B13] Dahnke R. Yotter R. A. Gaser C. (2013). Cortical thickness and central surface estimation. Neuroimage. 65, 336–348. 10.1016/j.neuroimage.2012.09.050 23041529

[B14] Damer C. K. Creutz C. E. (1994). Secretory and synaptic vesicle membrane proteins and their possible roles in regulated exocytosis. Prog Neurobiol. 43, 511–536. 10.1016/0301-0082(94)90051-5 7816934

[B15] Gao S. Chen Y. Sang F. Yang Y. Xia J. Li X.. (2019). White matter microstructural change contributes to worse cognitive function in patients with type 2 diabetes. Diabetes. 68, 2085–2094. 10.2337/db19-0233 31439643 PMC6804632

[B16] Gautam P. Anstey K. J. Wen W. Sachdev P. S. Cherbuin N. (2015). Cortical gyrification and its relationships with cortical volume, cortical thickness, and cognitive performance in healthy mid-life adults. Behav Brain Res. 287:331–339. 10.1016/j.bbr.2015.03.018 25804360

[B17] Goldman P. S. Galkin T. W. (1978). Prenatal removal of frontal association cortex in the fetal rhesus monkey: anatomical and functional consequences in postnatal life. Brain Res. 152, 451–485. 10.1016/0006-8993(78)91103-4 99206

[B18] Green S. Blackmon K. Thesen T. DuBois J. Wang X. Halgren E.. (2018). Parieto-frontal gyrification and working memory in healthy adults. Brain Imaging Behav. 12, 303–308. 10.1007/s11682-017-9696-9 28290070

[B19] Hanly J. G. Robertson J. W. Legge A. Kamintsky L. Aristi G. Friedman A.. (2022). Resting state functional connectivity in SLE patients and association with cognitive impairment and blood-brain barrier permeability. Rheumatology (Oxford). keac343. 10.1093/rheumatology/keac343 PMC9891437 35699463

[B20] Harreiter J. Roden M. (2019). Diabetes mellitus-Definition, classification, diagnosis, screening and prevention (Update 2019). Wien Klin Wochenschr. 131, 6–15. 10.1007/s00508-019-1450-4 30980151

[B21] Hsu J. L. Chen Y. L. Leu J. G. Jaw F. S. Lee C. H. Tsai Y. F.. (2012). Microstructural white matter abnormalities in type 2 diabetes mellitus: a diffusion tensor imaging study. Neuroimage. 59:1098–105. 10.1016/j.neuroimage.2011.09.041 21967726

[B22] Huang L. Chen X. Sun W. Chen H. Ye Q. Yang D.. (2020). Early segmental white matter fascicle microstructural damage predicts the corresponding cognitive domain impairment in cerebral small vessel disease patients by automated fiber quantification. Front Aging Neurosci. 12, 598242. 10.3389/fnagi.2020.598242 33505302 PMC7829360

[B23] Hutton C. Draganski B. Ashburner J. Weiskopf N. (2009). A comparison between voxel-based cortical thickness and voxel-based morphometry in normal aging. Neuroimage. 48:371–380. 10.1016/j.neuroimage.2009.06.043 19559801 PMC2741580

[B24] Ithapu V. Singh V. Lindner C. Austin B. P. Hinrichs C. Carlsson C. M.. (2014). Extracting and summarizing white matter hyperintensities using supervised segmentation methods in Alzheimer's disease risk and aging studies. Hum Brain Mapp. 35, 4219–4235. 10.1002/hbm.22472 24510744 PMC4107160

[B25] Kinno R. Mori Y. Kubota S. Nomoto S. Futamura A. Shiromaru A.. (2019). High serum high-density lipoprotein-cholesterol is associated with memory function and gyrification of insular and frontal opercular cortex in an elderly memory-clinic population. Neuroimage Clin. 22, 101746. 10.1016/j.nicl.2019.101746 30856540 PMC6411909

[B26] Lamballais S. Vinke E. J. Vernooij M. W. Ikram M. A. Muetzel R. L. (2020). Cortical gyrification in relation to age and cognition in older adults. Neuroimage. 212, 116637. 10.1016/j.neuroimage.2020.116637 32081782

[B27] Li W. Qin W. Liu H. Fan L. Wang J. Jiang T.. (2013). Subregions of the human superior frontal gyrus and their connections. Neuroimage. 78, 46–58. 10.1016/j.neuroimage.2013.04.011 23587692

[B28] Li W. Sun L. Li G. Xiao S. (2019). Prevalence, influence factors and cognitive characteristics of mild cognitive impairment in type 2 diabetes mellitus. Front Aging Neurosci. 11, 180. 10.3389/fnagi.2019.00180 31417393 PMC6682644

[B29] Li B. (2015). Human nature, the means-ends relationship, and alienation: themes for potential East–West collaboration. Technol Soc. 43, 60–64. 10.1016/j.techsoc.2015.03.005

[B30] Luders E. Thompson P. M. Narr K. L. Toga A. W. Jancke L. Gaser C.. (2005). curvature-based approach to estimate local gyrification on the cortical surface. Neuroimage. 29, 1224–1230. 10.1016/j.neuroimage.2005.08.049 16223589

[B31] Macpherson H. Formica M. Harris E. Daly R. M. (2017). Brain functional alterations in Type 2 Diabetes - a systematic review of fMRI studies. Front Neuroendocrinol. 47:34–46. 10.1016/j.yfrne.2017.07.001 28687473

[B32] Meng J. Liu J. Li H. Gao Y. Cao L. He Y.. (2022). Impairments in intrinsic functional networks in type 2 diabetes: a meta-analysis of resting-state functional connectivity. Front Neuroendocrinol. 66, 100992. 10.1016/j.yfrne.2022.100992 35278579

[B33] Nunez C. Callen A. Lombardini F. Compta Y. Stephan-Otto C. (2020). Alzheimer's Disease neuroimaging I. different cortical gyrification patterns in Alzheimer's disease and impact on memory performance. Ann Neurol. 88, 67–80. 10.1002/ana.25741 32277502

[B34] Petersen R. C. Lopez O. Armstrong M. J. Getchius T. S. D. Ganguli M. Gloss D.. (2018). Practice guideline update summary: mild cognitive impairment: report of the Guideline development, dissemination, and implementation subcommittee of the American academy of neurology. Neurology. 90, 126–135. 10.1212/WNL.0000000000004826 29282327 PMC5772157

[B35] Richman D. P. Stewart R. M. Hutchinson J. W. Caviness V. S. (1975). Mechanical model of brain convolutional development. Science. 189, 18–21. 10.1126/science.1135626 1135626

[B36] Rosenberg J. Lechea N. Pentang G. N. Shah N. J. (2018). What magnetic resonance imaging reveals - a systematic review of the relationship between type II diabetes and associated brain distortions of structure and cognitive functioning. Front Neuroendocrinol. (2019) 52, 79–112. 10.1016/j.yfrne.2018.10.001 30392901

[B37] Schaer M. Cuadra M. B. Tamarit L. Lazeyras F. Eliez S. Thiran J. P.. (2008). surface-based approach to quantify local cortical gyrification. IEEE Trans Med Imaging. 27, 161–170. 10.1109/TMI.2007.903576 18334438

[B38] Sterling N. W. Wang M. Zhang L. Lee E. Y. Du G. Lewis M. M.. (2016). Stage-dependent loss of cortical gyrification as Parkinson disease “unfolds”. Neurology. 86, 1143–1151. 10.1212/WNL.0000000000002492 26888982 PMC4820131

[B39] Uddin L. Q. (2015). Salience processing and insular cortical function and dysfunction. Nat Rev Neurosci. 16, 55–61. 10.1038/nrn3857 25406711

[B40] Van Essen D. C. (1997). A tension-based theory of morphogenesis and compact wiring in the central nervous system. Nature. 385, 313–318. 10.1038/385313a0 9002514

[B41] Wang Y. Zhang Y. Zhang J. Wang J. Xu J. Li J.. (2018). Structural and functional abnormalities of the insular cortex in trigeminal neuralgia: a multimodal magnetic resonance imaging analysis. Pain. 159, 507–514. 10.1097/j.pain.0000000000001120 29200179

[B42] Wardlaw J. M. Smith E. E. Biessels G. J. Cordonnier C. Fazekas F. Frayne R.. (2013). Neuroimaging standards for research into small vessel disease and its contribution to ageing and neurodegeneration. Lancet Neurol. 12, 822–838. 10.1016/S1474-4422(13)70124-8 23867200 PMC3714437

[B43] Xue M. Xu W. Ou Y. N. Cao X. P. Tan M. S. Tan L.. (2019). Diabetes mellitus and risks of cognitive impairment and dementia: a systematic review and meta-analysis of 144 prospective studies. Ageing Res Rev. 55, 100944. 10.1016/j.arr.2019.100944 31430566

[B44] Yan C. G. Wang X. D. Zuo X. N. Zang Y. F. (2016). DPABI data processing and analysis for (resting-state) brain imaging. Neuroinformatics. 14, 339–351. 10.1007/s12021-016-9299-4 27075850

[B45] Yang D. Qin R. Chu L. Xu H. Ni L. Ma J.. (2022). Abnormal cerebrovascular reactivity and functional connectivity caused by white matter hyperintensity contribute to cognitive decline. Front Neurosci. 16, 807585. 10.3389/fnins.2022.807585 35310084 PMC8930816

[B46] Zhang D. Wang M. Gao J. Huang Y. Qi F. Lei Y.. (2021). Altered functional connectivity of insular subregions in Type 2 diabetes mellitus. Front Neurosci. 15, 676624. 10.3389/fnins.2021.676624 34220433 PMC8242202

[B47] Zhang J. Wang Y. Wang J. Zhou X. Shu N. Wang Y.. (2014). White matter integrity disruptions associated with cognitive impairments in type 2 diabetic patients. Diabetes. 63, 3596–3605. 10.2337/db14-0342 24947353

[B48] Zhang Z. Zhang B. Wang X. Zhang X. Yang Q. X. Qing Z.. (2019). Olfactory dysfunction mediates adiposity in cognitive impairment of type 2 diabetes: insights from clinical and functional neuroimaging studies. Diabetes Care. 42, 1274–1283. 10.2337/dc18-2584 31221697

[B49] Zilles K. Palomero-Gallagher N. Amunts K. (2013). Development of cortical folding during evolution and ontogeny. Trends Neurosci. 36:275–284. 10.1016/j.tins.2013.01.006 23415112

