# Supplementary material for: Altered local gyrification and functional connectivity in type 2 diabetes mellitus patients with mild cognitive impairment: A pilot cross-sectional small-scale single center study
Source: Front Aging Neurosci. 2022 Sep 20;14:934071. doi: 10.3389/fnagi.2022.934071 (PMC9530449; doi:10.3389/fnagi.2022.934071)
Supplement: Supplementary file 1 [file Data_Sheet_1.docx]

**Table s1.** Raw scores of each cognitive test for participants of three groups.

|  | HC (n=167) | T2DM-NC (n=154) | T2DM-MCI (n=126) | F value | P-value |
| --- | --- | --- | --- | --- | --- |
| MMSE | 28.67±1.41 | 28.46±1.56 | 27.57.62±2.11 | 38.757 | ＜0.001 ^a,b^ |
| MOCA | 26.90±1.36 | 26.89±1.02 | 23.10±1.54 | 175.975 | ＜0.001 ^a,b^ |
| AVLT-LTDR | 5.54±2.35 | 4.66±2.26 | 3.31±2.46 | 27.497 | ＜0.001 ^a,b^ |
| VR-DR | 8.15±3.79 | 8.28±3.62 | 5.37±4.04 | 22.033 | ＜0.001 ^a,b^ |
| VRC | 13.64±2.36 | 13.32±1.64 | 12.48±3.53 | 7.744 | 0.001 ^a,b^ |
| CDT | 3.71±0.65 | 3.76±0.55 | 3.31±1.03 | 12.258 | ＜0.001 ^a,b^ |
| TMT-A | 52.11±24.10 | 54.15±29.58 | 88.91±72.42 | 28.825 | ＜0.001 ^a,b^ |
| TMT-B | 101.14±55.04 | 110.33±65.32 | 160.18±87.62 | 28.528 | ＜0.001 ^a,b^ |
| SCWT-B | 20.63±8.10 | 22.30±8.59 | 29.52±16.98 | 22.715 | ＜0.001 ^a,b^ |
| SCWT-C | 31.21±12.79 | 32.55±13.30 | 43.81±29.39 | 17.338 | ＜0.001 ^a,b^ |
| CVFT | 17.77±4.45 | 17.14±5.14 | 14.41±4.40 | 18.786 | ＜0.001 ^a,b^ |
| BNT | 51.90±5.45 | 51.90±8.66 | 45.65±10.58 | 23.212 | ＜0.001 ^a,b^ |

P < 0.05 had statistical significance. ^a^ Compare HC group to T2DM-MCI group, ^b^ Compare T2DM-NC group to T2DM-MCI group. Data are presented as mean±standard deviation (SD).

Abbreviations: *MMSE* Mini-Mental State Examination, *MoCA* Montreal Cognitive Assessment, *AVLT-LTDR* the auditory verbal learning test-long time delay recall test, *VR-DR* the visual reproduction-delay recall test, *VRC* the VR-copy test, *CDT* the clock drawing test, *TMT* the trail making test, *SCWT* the stroop color and word test, *CVFT* the category verbal fluency test, *BNT* the Boston naming test.


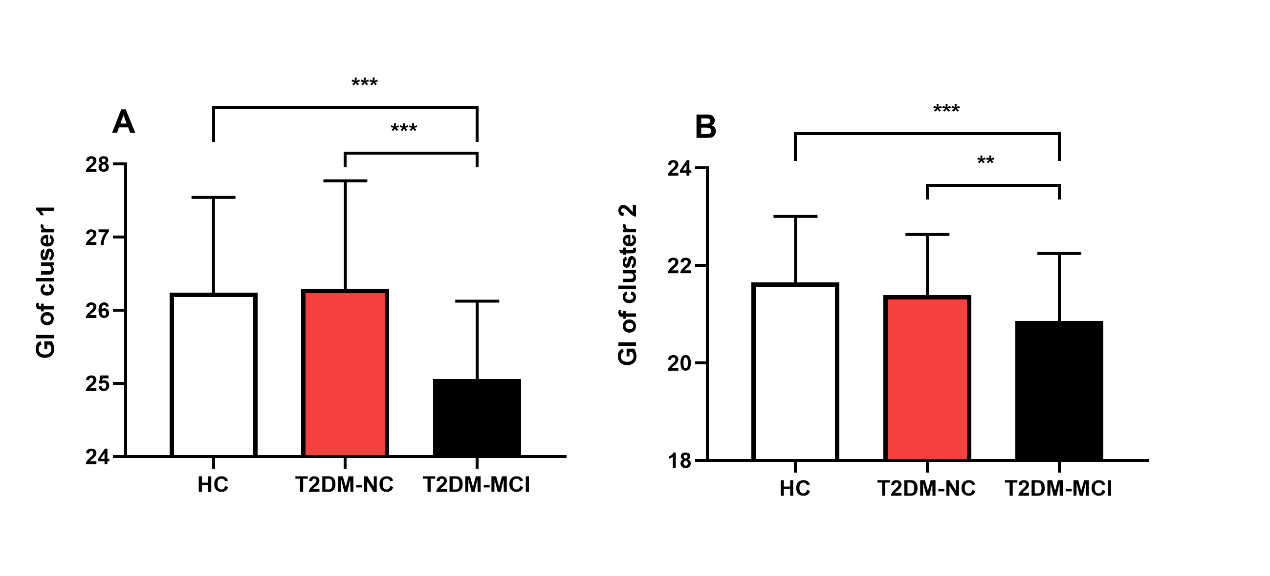


**Figure s1. Post-hoc analysis of clusters showing significant between-group differences in GI analysis. ^**^** indicates p < 0.01, **^***^** indicates p < 0.001. (Bonferroni corrected).


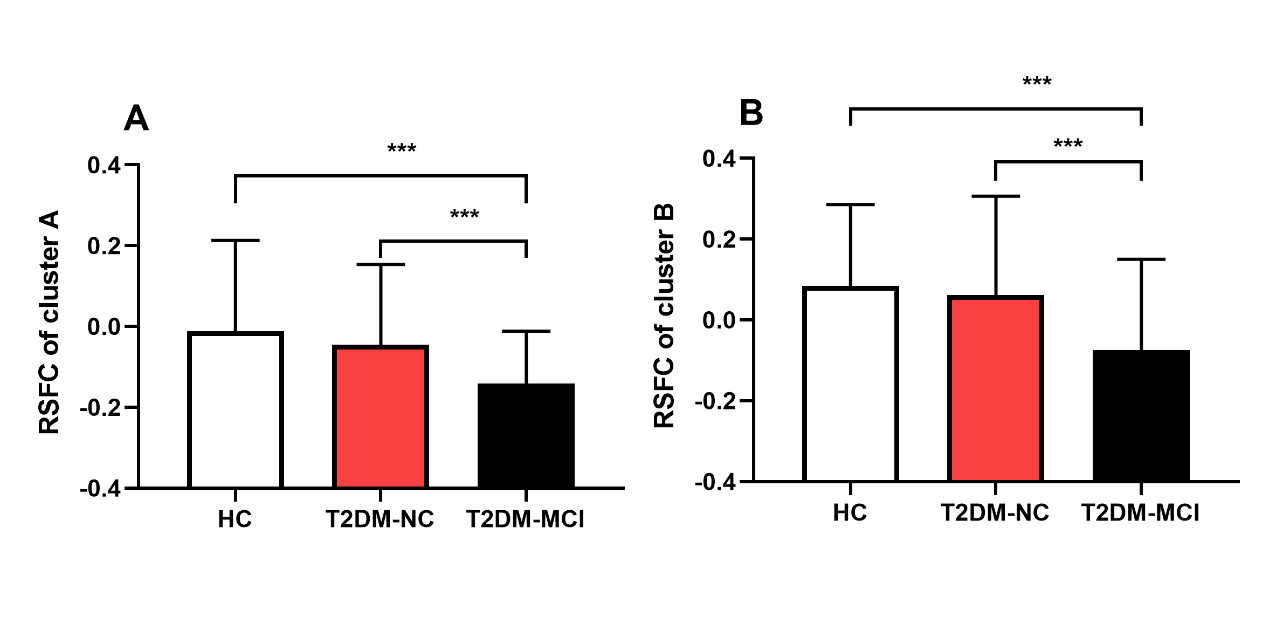


**Figure s2. Post-hoc analysis of clusters showing significant between-group differences in RSFC analysis. ^***^** indicates p < 0.001. (Bonferroni corrected).
